# Improved outcomes and reduced medical costs through multidisciplinary co-management protocol for geriatric proximal femur fractures: a one-year retrospective study

**DOI:** 10.1186/s12877-022-03014-6

**Published:** 2022-04-11

**Authors:** Yang Li, Kuan-Kai Tung, Yi-Cheng Cho, Shih-Yi Lin, Cheng-Hung Lee, Chih-Hui Chen

**Affiliations:** 1 Department of Orthopedics, Taichung Veterans General Hospital, Taichung, Taiwan; 2 School of Medicine, National Yang Ming Chiao Tung University, Taipei, Taiwan; 3 Center for Geriatrics and Gerontology, Department of Internal Medicine, Taichung Veterans General Hospital, Taichung, Taiwan; 4 Division of Endocrinology and Metabolism, Department of Internal Medicine, Taichung Veterans General Hospital, Taichung, Taiwan; 5 Department of Orthopedics, Changhua Christian Hospital, Changhua, Taiwan; 6 No. 135, Nanxiao St, Changhua County 50006 Changhua City, Taiwan

**Keywords:** Elderly hip fractures, Clinical pathway, Co-managed orthogeriatric care, Mortality, Medical expense

## Abstract

**Background:**

To manage the rapidly growing incidence of, and related medical burden resulting from hip fractures in older adults in an aging society, studies involving orthogeriatric co-management treatment models have reported improved outcomes, including reduced medical costs. The treatment gap for osteoporosis was however seldom emphasized in the published treatment protocols. Aiming to improve the existing orthogeriatric protocol, we have established a patient-centered protocol for elderly patient hip fractures, which simultaneously focuses on fracture care and anti-osteoporosis agent prescription in regarding to healthcare quality and medical expense.

**Methods:**

This was a retrospective study comparing patients who enrolled in the multidisciplinary co-managed protocol for geriatric hip fractures and those who did not. The inclusion criteria for this study were: (a) single-sided hip fractures treated from 1 to 2018 to 30 June 2020, (b) patients who were 60-years or older (c) trauma treated within 3 days from time of injury, and (d) minimal follow-up period of 12 months after surgery.

**Results:**

From 1 to 2018 to 30 June 2020, 578 patients were included (267 patients in the protocol group vs. 331 patients in the conventional group). The protocol group was associated with significantly reduced lengths of hospital stay (*p* = 0.041), medical expenditures (*p* = 0.006), and mortality (*p* = 0.029) during their acute in-hospital admission period. Early osteoporosis diagnosis and anti-osteoporosis agent prescription were achieved in the protocol group, with a significantly wider coverage for BMD assessment (*p* < 0.001) and prescriptions for anti-osteoporosis medication (*p* < 0.001). Yet, there was no significant decline in the one-year refracture rate in the protocol group.

**Conclusions:**

The implementation of a multidisciplinary co-managed care protocol for geriatric proximal femur fractures successfully improved patient outcomes with significantly reduced lengths of stay, medical expenditures, and mortality during the acute in-hospital admission period. The high prescription rate of anti-osteoporosis medication after hip fractures in the protocol group was not associated with a significantly lower re-fracture rate in the 12-month follow-up. However, the association between early anti-osteoporosis agent prescription and reduced long-term medical expenses in this group of patients has provided a direction for future research.

## Introduction

Hip fractures are a common and harmful injury in older adults. Despite its controlled incidence in developed countries, the increasing total number of elderly hip fractures in an aging society still has an impact on public health and the socio-economic system [1–4]. Although large improvements in pharmacology, surgical implants, and anesthetic management have been achieved in recent decades, hip fractures amongst the elderly population are consistently associated with significant mortality, morbidity, poor function and financial burden [4–7]. To manage the rapidly growing incidence of, and related burden from hip fractures in the elderly, studies on orthogeriatric co-management treatment models have reported improved outcomes with reduced lengths of hospital stays and medical costs [8]. Reviews regarding different care models have also been published in literature [9]. Both early diagnosis and treatment for osteoporosis however were seldom emphasized and integrated in treatment protocols for geriatric hip fractures. As osteoporosis is highly prevalent in patients with fragility hip fractures, the treatment gap for osteoporosis is positively associated with a high risk of refracture, which accounts for extra burden on public health [10]. The National Osteoporosis Foundation (NOF) recommends pharmacologic treatment in postmenopausal women and men with a personal history of hip or vertebral fracture, a T-score of − 2.5 or less, or a combination of low bone mass (T-score between − 1 and − 2.5) and a 10-year probability of hip fracture of at least 3% or any major fracture of at least 20% as calculated by the FRAX WHO Fracture Risk [11]. Aiming to reduce and alleviate mortality, comorbidity, and medical burden, we have thus established a patient-centered protocol for proximal femur fractures in the elderly, which not only aims to ameliorate healthcare quality during the acute in-hospital period, but also focuses on osteoporosis treatment in this patient population. This study evaluates the protocol’s effectiveness in outcome improvement and cost reduction, and represents an early result upon completion of the minimum one-year follow-up period. Moreover, with accordance to NOF recommendation on pharmacologic treatment with anti-osteoporosis medication,[11] association between early prescription of anti-osteoporosis agent and long-term medical cost was also inspected.

## Materials and methods

 Approved by the Ethics Committee of our institute, and with informed consent from the patients, this retrospective study was conducted in Taichung Veterans General Hospital (TCVGH, Taichung, Taiwan). The study compares patients who enrolled in the multidisciplinary co-managed protocol, introduced since 1 December 2018, for geriatric proximal femur fractures and those who did not, namely the protocol group and the conventional group, respectively. Several parameters were compared, including length and cost of acute in-hospital stay, time window from emergency unit to surgery, coverage of bone mineral densitometry (BMD) assessment and osteoporosis treatment, re-fracture rate, one-year returns and costs for outpatient, inpatient, emergency department, and overall medical services, in-hospital mortality rate, as well as one-year mortality rate. The medical costs were obtained as real-world data of the reimbursement declared to Taiwan National Health Insurance (NHI), which covers the expense from medication, examination, ward admission, surgery, and related medical appliances during hip fracture treatment, and directly reflects the burden of hip fractures on socio-economic system. The inclusion criteria for the study were: (a) single-sided proximal femur fractures treated surgically, (b) patients who were 60-years or older (c) trauma treated within 3 days from time of injury, and (d) a minimal follow-up period of 12 months after surgery. Subjects were excluded in case of multiple traumas, pathologic, periprosthetic or atypical fractures. In order to minimize the confounding effect of subjects’ baseline variables, the inclusion and exclusion criteria for the conventional group was set to correspond to those of the multidisciplinary co-managed protocol. Patients in the protocol group were included from 1 to 2018 to 30 June 2020, whereas in the conventional group, included patients were from 1 to 2018 to 30 June 2020. The inclusion period for conventional group was set a year before initiation of the co-management protocol to conduct the before and after comparisons. However, after the implementation on 1 December 2018, a small portion of patients requested to be operated by surgeons who did not take part in this protocol. Hence, they were treated under conventional care model, and not included in the protocol group. With most of the parameters being collected from medical records in a retrospective fashion, the one-year mortality for patients in the protocol group was obtained through phone surveys.

### The protocol for geriatric hip fracture

This multidisciplinary co-managed care protocol for geriatric patient fractures is comprised of many forms of expertise, including emergency units, orthopedic surgeons, geriatricians, anesthetists, cardiologists, rehabilitation physicians, neurologists, osteoporosis treatment centers, post-acute care referral teams, and nursing teams. It is a patient-centered care protocol, aiming to reduce mortality and comorbidity, improve functional outcomes and quality of life, and reduce overall medical expenditures for geriatric patient hip fractures.

**Fig. 1:**
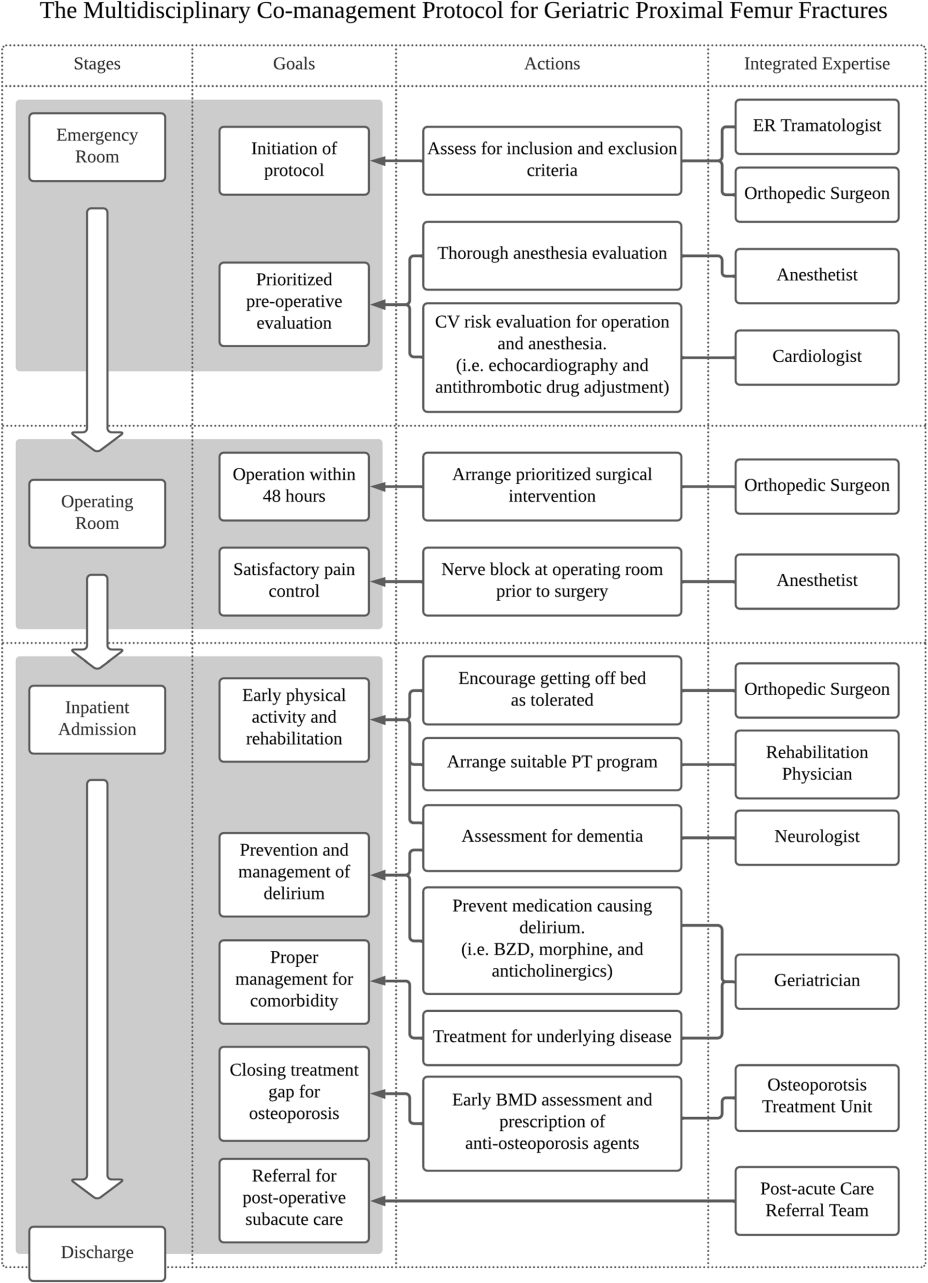
The summarized clinical pathways in the multidisciplinary co-management protocol for geriatric proximal femur fractures

Beginning from a patient’s arrival at the ER, a consulting orthopedic surgeon will provide an assessment for initiation of the protocol. As Fig. 1 illustrates, once surgical intervention is indicated and protocol initiated, a thorough pre-operative evaluation including anesthesia and cardiovascular risk is organized by anesthetists and cardiologists. Prioritized surgical intervention is then arranged, with an aim to perform surgery within 48 h upon patient’s arrival at the emergency room. Pain control protocol involving nerve block is performed by anesthetists prior to surgery. Four assigned senior orthopedic surgeons are responsible for the operations. The post-operative care and treatment for underlying comorbidities are managed in a co-ownership fashion, with one orthopedic attending and one geriatrician attending. Integration involving a rehabilitation physician and neurologists includes early physical activity and rehabilitation. The avoidance of benzodiazepines, narcotics, and anticholinergics lessens any incidence of delirium during the in-hospital stay. Through the Osteoporosis Treatment Center, both early diagnosis and treatment for osteoporosis are achieved. Additionally, patients are educated with the updated information from guidelines and recommendations from NOF. Options for anti-osteoporosis medication in our facility include bisphosphonates, Denosumab, Raloxifene, Teriparatide, and Romosozumab, which are prescribed according to physicians’ preferences when criteria established by Taiwan NHI are met. For unreimbursed patients who did not meet the criteria, the anti-osteoporosis agent can still be prescribed at one’s own expense. All the patients receiving anti-osteoporotic agents, also received calcium and vitamin D supplements. Subsequently, if needed, the post-acute referral team will be responsible for any required referral to other medical facilities for subacute care.

After discharge, patients will return to an integrated outpatient clinic consisting of orthopedic surgeon, rehabilitation physician, neurologist, and geriatrician, in order to facilitate the coordination of treatments and medications from different subspecialities.

### Anti-osteoporosis agent prescription

To further investigate the association of early anti-osteoporosis agent prescription with long-term medical visits and expenditure in elderly patients with hip fractures, the included patients were reallocated into two groups depending on the timing that anti-osteoporosis agent was prescribed. Patients whose prescription were initiated within one month post discharge were categorized into patient group on anti-osteoporosis agent, otherwise into patient group not on anti-osteoporosis agent. Parameters with regard to 1-year visits and medical expenditures on outpatient clinic, inpatient admission, emergency department, and overall medical were compared between these two groups.

### Statistics

Statistical analysis was carried out using IBM SPSS version 22.0 (International Business Machine Corp, New York, USA). Continuous data were analyzed with the Mann-Whitney test, and categorial data were analyzed using the Chi-square test.

## Results

From 1 to 2018 to 30 June 2020, 578 patients were included, amongst which 267 were enrolled in the multidisciplinary co-management care protocol, with 311 treated in a conventional care model. The baseline characteristics of these two groups of patients are compared and listed in Table 1.

**Table 1:** Baseline characteristics and demographic data of conventional group and protocol group

	Conventional group (*n *= 311)		Protocol group (*n* = 267)		*p* value
**Age**	79	± 9	82	± 9	< 0.001**
**Gender**					
Male (№)	109	(35.0%)	115	(43.1%)	0.059
Female (№)	202	(65.0%)	152	(56.9%)	
**Diagnosis**					< 0.001**
Femoral neck fracture	187	(60.1%)	165	(61.8%)	
Intertrochanteric fracture	95	(30.5%)	100	(37.5%)	
ITF with subtrochanteric extension	29	(9.3%)	2	(0.7%)	
**Osteoporosis**					
BMD < -2.5 (*n* = 352)	82/142	(57.7%)	119/210	(56.7%)	0.927
**Comorbidities**					
Urology	10	(3.2%)	19	(7.1%)	0.051
Nephrology	38	(12.2%)	38	(14.2%)	0.555
Cardiology	131	(42.1%)	124	(46.4%)	0.338
Pulmonology	31	(10.0%)	27	(10.1%)	1.000
Gastroenterology	19	(6.1%)	20	(7.5%)	0.621
Neurology	27	(8.7%)	19	(7.1%)	0.590
DM	72	(23.2%)	64	(24.0%)	0.894

ITF, Intertrochanteric fracture

Mann-Whitney test. Chi-Square test. *p < 0.05, **p < 0.01

Continuous data were expressed with mean ± SD

Categorial data were expressed with number and percentage

The comparisons between the two groups are presented in Table 2. During the short-term acute in-hospital period, a significantly reduced length of hospital stay was seen in the protocol group (*p* = 0.041). This was similar to medical expenditures for acute in-hospital admission in the protocol group, which was significantly reduced by 29.58% on average. (*p* = 0.006)

**Table 2:** Intergroup comparison of clinical outcomes, medical expenses, mortality rates, and coverage for early osteoporosis diagnosis and treatment

	Conventional group (*n* = 311)		Protocol group (*n* = 267)		*p* value	
**Acute in-hospital stay**						
Length of hospital stay (day)	11.9	± 12.1	11.6	± 10.5	0.041*	
Medical expenditure (NTD)	163,269	± 161,522	135,503	± 117,206	0.006*	
**Time to surgery**						
Window from ER to OR (hrs)	46	± 93	34	± 43	0.059	
Time to surgery > 48 h (№)	59	(20.6%)	42	(15.8%)	0.174	
**Osteoporosis diagnosis and anti-osteoporosis agent prescription**						
BMD assessment (№)	163	(52.4%)	253	(94.8%)	< 0.001**	
Prescription of anti-osteoporosis medication (№)	78	(25.1%)	155	(58.1%)	< 0.001**	
**Re-fracture (**№**)**	38	(12.2%)	32	(12.0%)	1.000	
**Returns for overall medical services (1-year)**						
Number of patients returned (№)	263	(84.6%)	236	(88.4%)	0.225	
Average expenditure per returned patient (NTD)	131,766	± 237,171	114,001	± 219,625	0.621	
Average expenditure per patient in treatment group (NTD)	111,429	± 223,190	100,765	± 209,649	0.692	
**Returns for outpatient clinic (1-year)**						
Number of patients returned (№)	251	(80.7%)	226	(84.6%)	0.257	
Average visits per returned patient (times)	13	± 12	13	± 13	0.448	
Average expenditure per returned patient (NTD)	55,079	± 124,811	38,121	± 52,255	0.261	
Average expenditure per patient in treatment group (NTD)	44,453	± 114,178	32,267	± 49,993	0.096	
**Returns for emergency department (1-year)**						
Number of patients returned (№)	83	(26.7%)	74	(27.7%)	0.855	
Average visits per returned patient (times)	2	± 2	2	± 2	0.973	
Average expenditure per returned patient (NTD)	21,620	± 24,358	22,184	± 25,092	0.502	
Average expenditure per patient in treatment group (NTD)	5770	± 15,770	6148	± 16,485	0.694	
**Returns for inpatient admission (1-year)**						
Number of patients returned (№)	78	(25.1%)	57	(21.3%)	0.338	
Average visits per returned patient (times)	2	± 2	2	± 1	0.815	
Average expenditure per returned patient (NTD)	244,042	± 267,379	292,056	± 300,443	0.634	
Average expenditure per patient in treatment group (NTD)	61,207	± 170,248	62,349	± 182,700	0.331	
**Mortality**						
In-hospital mortality	18	(5.8%)	5	(1.9%)	0.029*	
1-year mortality	N/A		31	(11.6%)	N/A	

Mann-Whitney test. Chi-Square test. *p < 0.05, **p < 0.01

Continuous data were expressed with mean ± SD

Categorial data were expressed with number and percentage

All the parameters regarding 1-year medical visits and expenditure in either overall, outpatient clinic, emergency department, and inpatient admission sections were not significantly different between groups. Lower average expenditure per returned patient and per patient in group for overall medical expenditure were however observed in protocol group, although this was not statistically significant.

With prioritized surgical intervention, the protocol group was associated with a reduced time to surgery interval, dropping from 46 to 34 h on average. The fraction of patients who were operated on beyond 48 h was also reduced from 20.6 to 15.8% with the protocol. Although in these parameters, neither improvement was statistically significant.

Both early osteoporosis diagnosis and treatment were achieved in the protocol group. The coverage of BMD assessment through Dual X-ray Absorptiometry (DXA) and osteoporosis medication administration improved from 52.4 to 94.8% (*p* < 0.001) and 25.1 to 58.1% (*p* < 0.001), respectively. Such amelioration represents the closure of the treatment gap for osteoporosis.

After discharge, 84.6% of patients in the conventional group and 88.4% of patients in the protocol group returned at least once for medical services within a one-year period. Yet there existed no significant difference between groups. Likewise for the parameters regarding long-term medical expenditures and visits, the difference between the protocol group and conventional group was insignificant.

The imminent risk of refracture is high for patients not on anti-osteoporosis agents after hip fractures. Our results however showed no decline in the refracture rate associated with implementation of the protocol.

In terms of patient survival, the in-hospital mortality rate was 1.9% in the protocol group, which is significantly lower than 5.8% in the conventional group. (*p* = 0.029) The one-year mortality rate in the protocol group was obtained through phone interviews conducted during May and June of 2021, and was 11.6% amongst the patients who had enrolled in the protocol.

**Table 3:** Relation of early anti-osteoporosis agent prescription with 1-year medical visits and expenditure. Associations between early prescription of anti-osteoporosis agents and long-term medical costs were analyzed and presented in Table 3. A total of 233 patients had their anti-osteoporosis agent prescribed within one month post discharge, and 345 patients had not. Both the number of patients returned for overall medical services and the average expenditure (per returned patient or per patient in treatment group) for overall medical services were significantly lower in patient on anti-osteoporosis agent (*p* < 0.001, *p* = 0.005, and *p* < 0.001, respectively), whilst average expenditure (per returned patient or per patient in treatment group) for outpatient clinic service was significantly higher in patient group on anti-osteoporosis agent (*p* < 0.001 and *p* < 0.001 respectively), as compared to patient group not on anti-osteoporosis agent

	Patients not on anti-osteoporosis agent (*n* = 345)		Patients on anti-osteoporosis agent (*n* = 233)		*p* value	
**Returns for overall medical services (1-year)**						
Number of patients returned (№)	283	(84.6%)	216	(88.4%)	< 0.001**	
Average expenditure per returned patient (NTD)	132,500	± 258,635	111,394	± 182,922	0.005**	
Average expenditure per patient in treatment group (NTD)	108,688	± 239,649	103,267	± 178,470	< 0.001**	
**Returns for outpatient clinic (1-year)**						
Number of patients returned (№)	269	(78.0%)	208	(89.3%)	< 0.001**	
Average visits per returned patient (times)	13	± 13	13	± 12	0.036*	
Average expenditure per returned patient (NTD)	45,079	± 114,539	49,585	± 70,357	< 0.001**	
Average expenditure per patient in treatment group (NTD)	35,149	± 102,815	44,265	± 68,215	< 0.001**	
**Returns for emergency department (1-year)**						
Number of patients returned (№)	91	(26.4%)	66	(28.3%)	0.673	
Average visits per returned patient (times)	2	± 2	2	± 2	0.332	
Average expenditure per returned patient (NTD)	21,865	± 24,092	21,915	± 25,536	0.957	
Average expenditure per patient in treatment group (NTD)	5767	± 15,652	6208	± 16,752	0.605	
**Returns for inpatient admission (1-year)**						
Number of patients returned (№)	83	(24.1%)	52	(22.3%)	0.700	
Average visits per returned patient (times)	2	± 2	2	± 1	0.779	
Average expenditure per returned patient (NTD)	281,704	± 303,361	236,559	± 243,513	0.408	
Average expenditure per patient in treatment group (NTD)	67,772	± 190,991	52,794	± 150,927	0.546	

Mann-Whitney test. Chi-Square test. *p < 0.05, **p < 0.01

Continuous data were expressed with mean ± SD

Categorial data were expressed with number and percentage

## Discussion

This study demonstrated that integrated care models significantly improve outcomes for geriatric patients with proximal femur fractures, including length of stay in acute hospital care, medical expenditures in acute hospital stay, and in-hospital mortality rates (Table 2).

A significantly shortened average length of stay (11.6 ± 10.5 vs. 11.9 ± 12.1 days, *p* = 0.041), with a concurrent cost-down effect (135,503 ± 117,206 vs. 163,269 ± 161,522 NTD, *p* = 0.006) during the acute in-hospital period was observed amongst the patients who were enrolled in the protocol, in comparison to patients treated conventionally. These findings are compatible with results from similar reports [12]. In our data, long-term medical expenditures for overall and outpatient visits were lower in protocol group (111,429 ± 223,1901 vs. 100,765 ± 209,649 NTD and 44,453 ± 114,178 vs. 32,267 ± 49,993 NTD), although without any significance (*p* = 0.692 and *p* = 0.096, Table 2). This may be secondary to the large dispersion of data having a large standard deviation due to unremoved outliers. Moreover, a higher coverage for anti-osteoporosis agent in the protocol group also signifies an increased expenditure from costly anti-osteoporosis medications (The coverage of osteoporosis medication is 58.1% vs. 25.1%, *p* < 0.001). Convincingly, despite the extra cost, average expenditure per patient in treatment group to the outpatient clinic was still lower in the protocol group. The reduced long term medical expenditures can be explained by the co-management setting of our protocol, in that early and prompt interventions were taken for any underlying diseases and comorbidities during the in-hospital period. The integrated clinician also facilitated the coordination of treatments from the different subspecialities, which concurrently prevents harmful drug interactions and prohibits waste from repetitive prescriptions. Simultaneously, early diagnosis for osteoporosis and prompt anti-osteoporosis agent prescription may also have a role in reducing long-term medical expenditure.

The economic burden of geriatric patient proximal femur fractures has been emphasized during the past two decades [4, 13, 14]. The multidisciplinary co-managed care protocol for geriatric patient proximal femur fractures in our study can potentially be one of the solutions to ease such a burden. Nonetheless, longer observation periods and larger sample sizes are still warranted in order to provide a solid confirmation of this conclusion.

There have been studies reporting ameliorated in-hospital mortality, with or without significance, through multidisciplinary co-managed protocols regarding hip fractures [9, 15–19]. One study which reviewed in-hospital mortality rates in the available literature, discovered rates ranging from 2.7 to 15% amongst patients with surgically treated proximal femur fractures who were treated in conventional care, and rates from 0.6 to 6% amongst those in the orthogeriatric co-management model [9]. The in-hospital mortality rate was significantly reduced from 5.8 to 1.9% with our protocol (*p* = 0.029). The one-year mortality rate for the protocol group was 11.6%, which did not outstrip the rates from published studies that had implemented similar co-management protocols [9, 19]. Reduced one-year mortality upon the implementation of integrated protocol for geriatric hip fractures was concluded in multiple studies [9]. Such improvement however remains inconclusive due to inaccessible one-year mortality rates for the conventional group from the national census registry.

In our study, the average time interval from emergency unit to surgery improved from 46 to 34 h with the protocol in place, although the improvement was insignificant. Similarly, the portion of patients who received surgery beyond 48 h was reduced, also in absence of statistical significance. These results were possibly sequential to the rather ordinarily short emergency-to-surgery interval occurring in our facility. The average 46-hour interval to surgery in our facility for the conventional group is rather short, as compared to the average 45.8 to 76.8 h in control groups in similar studies implementing hip fracture protocols [17, 20]. This somewhat explains the limited improvement, despite the implementation of our protocol.

Despite clear evidence that osteoporosis treatment reduces the risk of re-fractures and mortality in patients suffering from fragility fractures, the treatment gap remained considerable [21]. A systemic review and meta-analysis suggests that an orthogeriatric co-management model may expedite both the diagnosis of, and treatment for, osteoporosis in patients with fragility fractures. The lack of high-quality evidence in the reviewed literature however limits the credibility of such conclusion [22]. Heltne et al. stated that bisphosphonates were prescribed to 3.2% of patients under usual care versus 21.5% under geriatrician-led care [23]. A population-based cohort study showed that orthogeriatric co-management was associated with significantly increased prescription of osteoporosis medication at discharge [24]. Four other studies have reported an improved coverage of osteoporosis medication after orthogeriatric care implementation, rising from 30.9 to 54.6% [25], 11.8 to 68.9% [26], 1 to 54% [27], and 39.6 to 65.5%  [28]. Similarly in this study, coverage rate for early anti-osteoporosis agent prescription significantly improved from 25.1% in the conventional group to 58.1% in the protocol group (*p* < 0.001) Also, the coverage for early BMD assessment significantly improved from 52.4 to 94.8% through our protocol. (*p* < 0.001) Seemingly, there is still room for improvement with a 58.1% coverage rate for early anti-osteoporosis agent prescription in the protocol group. This rate was due to the strict reimbursement criteria for osteoporosis medication under the policy of Taiwan NHI, in which one must have a T-score less than − 2.5 and concurrently sustained from at least one episode of fragility fracture. The criteria for more expensive agents such as Teriparatide and Romosozumab are even stricter, that minimum requirements are a T-score less than − 3.5 with concurrent history of at least two fragility fractures. These strict criteria discourage patients with fragility fracture, but a borderline T-score, from undergoing treatment regimen for osteoporosis. A small proportion of patients was still willing to undergo unreimbursed treatment during the cohort, as recommended by NOF. In summary, not only did the multidisciplinary co-management care protocol reduce the treatment gap, it also helped achieve early diagnosis of osteoporosis. These two factors are crucial for patients with hip fragility fracture, as the condition is both a sign and a symptom of osteoporosis.

Furthermore, we have noticed that regardless of treatment pathways, the average one-year medical expenditures were significantly lower in the patient group on anti-osteoporosis agents (Table 3, *N* = 233), when compared to the patient group who are not. (*N* = 345, *p* = 0.005). On the contrary, the average expenditure for those in the outpatient clinic was significantly higher in the patient group on anti-osteoporosis agent (*p* < 0.001), which represents extra costs from anti-osteoporosis agents. This implies that a high prescription rate of anti-osteoporosis agent is associated with lower long-term medical expenditures in elderly patients with hip fractures, regardless of any extra costs incurred from pricy anti-osteoporosis medication.

Despite a higher prescription rate of anti-osteoporosis agent, the re-fracture rates of 12.2% in the conventional group and 12.0% in the protocol group are not significantly different. The cause of such insignificance can be multifactorial. Firstly, anti-osteoporosis treatment would come into effect after a certain period of adherence, ranging from 6 to 12 months in order to increase BMD, and an even longer period for re-fracture risk reduction [21, 29–32]. Secondly, patients’ compliances to medication and incidence of fall injuries might also influence the rate of refracture. Eventually, the incidence of re-fracture was recorded as real-world data from a single facility, which might have led to possible underestimation in either group.

The multidisciplinary approach to elderly hip fractures is an emerging concept in the recent decade, for improvement on patient outcomes was limited with advancement on surgical implants and techniques alone [5–7]. Various care protocols were proposed through different approaches, but all with similar goals. A multifacility study with sample size of 9,360 patients has demonstrated that standardized hospital-based hip fracture programs are effective in reducing incidence of perioperative medical and surgical complications, and are associated with a lower 30-day readmission rate, while having a significantly longer length of hospital stay. Longer length of stay from delayed discharge in exchange for patient optimization has yielded lower rates of discharge to an inpatient facility and 30-day readmission [33]. These comanaged, protocol-driven programs with standardized clinical pathways shares similar features with our protocol, and are all associated with improved short-term outcomes. The intention to discharge patient to home as opposed to other inpatient facilities is however not emphasized in our protocol. To implement such idea into our study, the correlation between medical costs, outcome measures, and discharge of patients to inpatient facility through post-acute care referral could be further investigated. Interestingly, similar care pathway incorporates intensivists into the multidisciplinary team, that pre-operative optimization and evaluation, and post-operative assessment and care all take place in Surgical Intensive Care Unit (SICU). They have achieved significant lower incidence of in-hospital complications (UTI, sepsis, acute renal failure, and decubitus ulcers) and shorter overall length of hospital stay in the pathway group, but similar in-hospital mortality rate between pre- and post-pathway groups [34]. The results are promising. However, in the setting of hip fractures in Taiwan, routine perioperative care involving intensivists in SICU would cost tremendously, and is not feasible under the regime of Taiwan NHI, in which policies are mostly public-health-based. Another prospective study has proposed an orthogeriatric acute hip fracture unit, where hip fracture patients were directly admitted from the emergency department to dedicated hip unit that provides orthogeriatric co-managed care. Although standardized approaches to fluid administration, pain management, thromboprophylaxis, and osteoporosis treatment were absent in the proposed care model, early and thorough preoperative geriatric assessment with daily medical support from geriatrician has accomplished significant improvements in time to surgery, length of stay, and length acute and post-acute stay [35]. In contrast to our study where medical supports from geriatrician begin after admission, the early geriatric assessment at pre-operation stage might provide immediate intervention for underlying diseases and other medical issues without delaying the surgery. Fast track admission before surgery to dedicated ward for early geriatrician support is potentially a point to improve to our protocol. Finally, a retrospective study has reviewed the effectiveness of standardized interdisciplinary hip fracture protocol on, specifically, patients with femoral neck fractures treated with hemiarthroplasties (HA) or total hip arthroplasties (THA). A significantly decreased rate of major complication, decreased median length of hospital stay, decreased 1-year mortality, and increased rate of discharge home were observed amongst patients treated after the introduction of the protocol [36]. Despite the fact that subgroup analysis with accordance to fracture patterns or types of operated surgeries was not performed in this study, the protocol-driven treatment pathways might provide different extents of benefit to patient associated with certain fracture pattern or surgical intervention. With future research, customized protocols for specific fracture patterns or surgical procedures could be adopted to further improve our protocol.

There exist several limitations in our study. First of all, the retrospective design of the study is prone to selection bias. Long-term medical expenditures and visits involved real-world data from a single medical center. Underestimations due to lost follow-up data could also act as potential biases. A larger sample size could reduce the effect of the underestimation, as it occurred in both the protocol and conventional groups. Also, the initial functional and mobility status before injury as well as patients’ residencies, which are known factors affecting outcomes of hip fractures, were not documented due to the retrospective nature of this study. Acquisition of these data could further eliminate their potential confounding effects. Withal, access to the national census registry could possibly provide data for long-term mortality analysis.

## Conclusions

The implementation of a multidisciplinary co-managed care protocol for geriatric proximal femur fractures successfully improved outcomes through significantly reduced lengths of stay, medical expenditures, and mortality rates, during the acute in-hospital admission period. The achieved improvements from this protocol alleviates the medical burden of elderly hip fractures on Taiwan NHI, hence mitigation of the load on socio-economic system. Although the higher prescription rate of anti-osteoporosis was not reflected by a reduced re-fracture rate in the protocol group, the association between early prescription of anti-osteoporosis medication and reduced long-term overall medical expenses has provided a future direction for further research.

## Data Availability

All data relevant to the study are included in the article or uploaded as supporting information. The datasets used during this study are available from the provided link: “https://docs.google.com/spreadsheets/d/1tlf5Iz5dw0YB6gfaJpmNm3R6hJ5huTdQ/edit?usp=sharing&ouid=117003647672348102124&rtpof=true&sd=true”. The link should be permanently accessible, please contact the corresponding author in case of expired link.
