# Correction: Improved outcomes and reduced medical costs through multidisciplinary co-management protocol for geriatric proximal femur fractures: a one-year retrospective study

**DOI:** 10.1186/s12877-022-03136-x

**Published:** 2022-06-14

**Authors:** Yang Li, Kuan-Kai Tung, Yi-Cheng Cho, Shih-Yi Lin, Cheng-Hung Lee, Chih-Hui Chen

**Affiliations:** 1 Department of Orthopedics, Taichung Veterans General Hospital, Taichung, Taiwan; 2 School of Medicine, National Yang Ming Chiao Tung University, Taipei, Taiwan; 3 Center for Geriatrics and Gerontology, Department of Internal Medicine, Taichung Veterans General Hospital, Taichung, Taiwan; 4 Division of Endocrinology and Metabolism, Department of Internal Medicine, Taichung Veterans General Hospital, Taichung, Taiwan; 5 Department of Orthopedics, Changhua Christian Hospital, Changhua, Taiwan; 6 No. 135, Nanxiao St, Changhua County, 50006 Changhua City, Taiwan

**Correction: BMC Geriatrics 22, 318 (2022)**

**https://doi.org/10.1186/s12877-022-03014-6**

After publication of this article [1], the authors reported that in this article the last paragraph of the Results section, erroneously has become part of the legend of Table 3.

The last paragraph of the Results section should read: “Associations between early prescription of anti-osteoporosis agents and long-term medical costs were analyzed and presented in Table 3. A total of 233 patients had their anti-osteoporosis agent prescribed within one month post discharge, and 345 patients had not. Both the number of patients returned for overall medical services and the average expenditure (per returned patient or per patient in treatment group) for overall medical services were significantly lower in patient on anti-osteoporosis agent (p < 0.001, p = 0.005, and p < 0.001, respectively), whilst average expenditure (per returned patient or per patient in treatment group) for outpatient clinic service was significantly higher in patient group on anti-osteoporosis agent (p < 0.001 and p < 0.001 respectively), as compared to patient group not on anti-osteoporosis agent.”

The table legend should read: “Table 3. Relation of early anti-osteoporosis agent prescription with 1-year medical visits and expenditure.”

The original article [1] has been updated.
